# Supplementary material for: Ultrabright Fluorescent Silica Nanoparticles for Multiplexed Detection
Source: Nanomaterials (Basel). 2020 May 8;10(5):905. doi: 10.3390/nano10050905 (PMC7279313; doi:10.3390/nano10050905)
Supplement: Supplementary file 1 [file nanomaterials-10-00905-s001.pdf]

Supplementary materials

for

Ultrabright fluorescent silica nanoparticles for multiplexed detection

Saquib Ahmed M. A. Peerzade[1], Nadezhda Makarova[2], and Igor Sokolov [1,2,3,*]

[1] Department of Biomedical Engineering, Tufts University, Medford, MA 02155, USA

[2] Department of Mechanical Engineering, Tufts University, Medford, MA 02155, USA

[3] Department of Physics, Tufts University, Medford, MA 02155, USA

* Correspondence: igor.sokolov@tufts.edu

Calculation of the FRET efficiency

The FRET efficiency (E) is given by,

$$E = \frac{1}{1+\left({}^{r}/_{R}\right)^{6}}. \quad \text{(S1)}$$

where, r is the distance between tbe dye molecules and R is the Förster radius, which can be calculated from the normalized fluorescence spectra of the donor and normalized molar extinction coefficient spectra of the acceptor using equation (S2)[1]:

$$R = \left(\frac{9000(\ln 10)\kappa^2 \emptyset_D}{128\pi^5 N n^4} \int_0^\infty F_D(\lambda)\varepsilon_A(\lambda)\lambda^4 d\lambda\right)^{\left({}^{1}/_{6}\right)}. \quad \text{(S2)}$$

where, κ^2 is the dipole orientation factor (κ^2=2/3 for the random distribution of dipole moments), $\emptyset_D$ the quantum yield of the donor, N the Avogadro number, n the refractive index of the medium, F_D the fluorescence intensity of the donor, and ε_A the extinction coefficient of the acceptor.

Table S1. DLS size measurements of the synthesized particles.

	**5 dyes**	**3 dyes**	**2 dyes molecular ratio 0.1:1**
**Z-average**	99 ± 0.7	75 ± 0.3	74 ± 1
**Number Mean**	76 ± 1	56 ± 2	57 ± 3
**PDI**	0.073	0.064	0.059

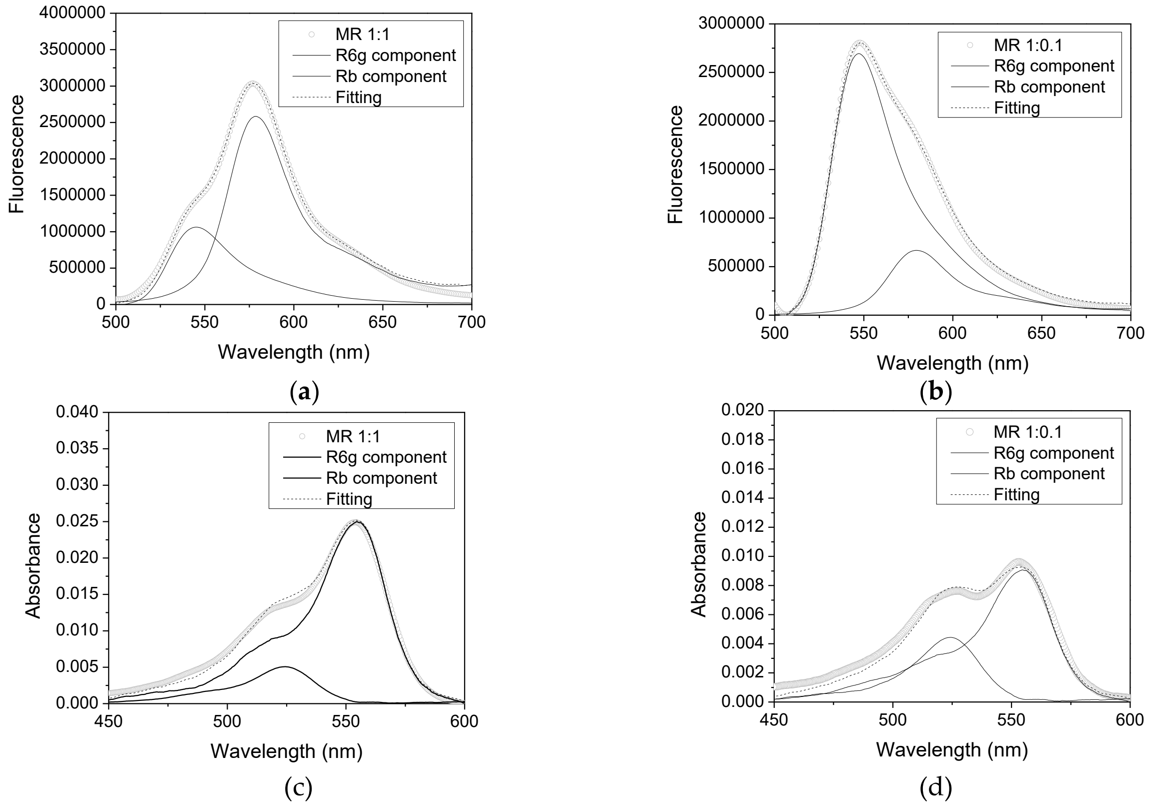

Figure S1. Fluorescence and absorbance spectra of Star dots with two encapsulated dyes of R6G and RB at different molar proportions. The individual components of the spectra corresponding to individual R6G and RB dyes are also shown (found using the linear decomposition algorithm described in the Method section).

The fluorescence spectra (a) MR1:1 and (b) MR1:0.1. The particles are excited with 488 nm. The absorbance spectra of Star dots (c) MR1:1 and (d) MR1:0.1 particles.

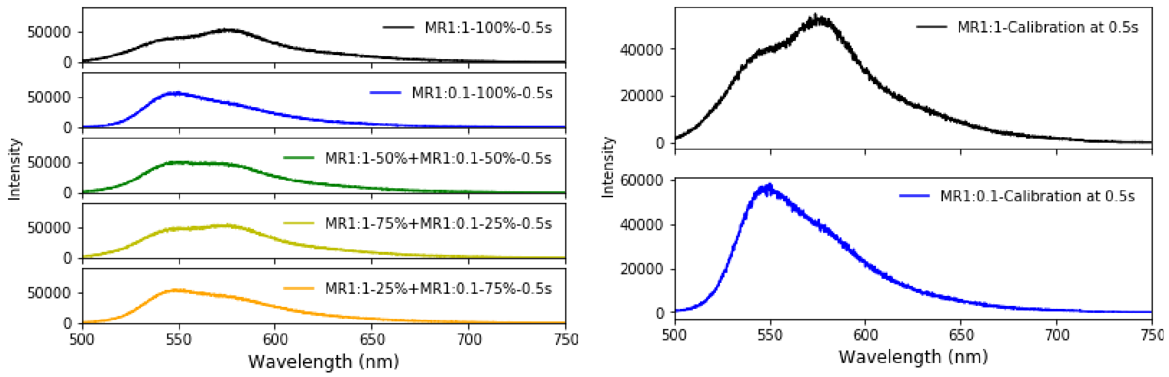

(**a**) (**b**)

Figure S2. Verification of multiplexing. (**a**) Fluorescence spectra collected with 100% of MR1:1 and MR1:0.1 and mix of 50+50, 75+25, 25+75% mix of MR1:1+MR1:0.1 particles respectively, excited at 488 nm. The time of collection of the fluorescence spectrum was 0.5 sec. (**b**) Spectra of pure MR1:1 and MR1:0.1 particles collected for 0.5 sec.

Table S2. Demultiplexing using different mixes of MR1:1 and MR1:0.1 particles excited at 488 nm and collected for 0.5sec.

Actual mix % MR1:1 : MR1:0.1 particles	Calculated mix % of MR1:1 : MR1:0.1	Error in demultiplexing MR1:1 / MR1:0.1	Average error in demultiplexing
100:0	100:0	0/0	0
25:75	29:74	4/1	3
50:50	47:55	3/5	4
75:25	79:29	4/4	4
0:100	0:100	0/0	0

Table S3. Demultiplexing using different mixes of MR1:1 and MR1:0.1 particles excited at 488 nm and collected for 0.5sec with a weight function proportional to the fluorescent intensity.

Actual mix % MR1:1 : MR1:0.1 particles	Calculated mix % of MR1:1 : MR1:0.1	Error in demultiplexing MR1:1 / MR1:0.1	Average error in demultiplexing
100:0	100:0	0/0	0
25:75	27:75	2/0.4	1
50:50	47:55	3/5	4
75:25	79:29	3/5	4
0:100	0:100	0/0	0

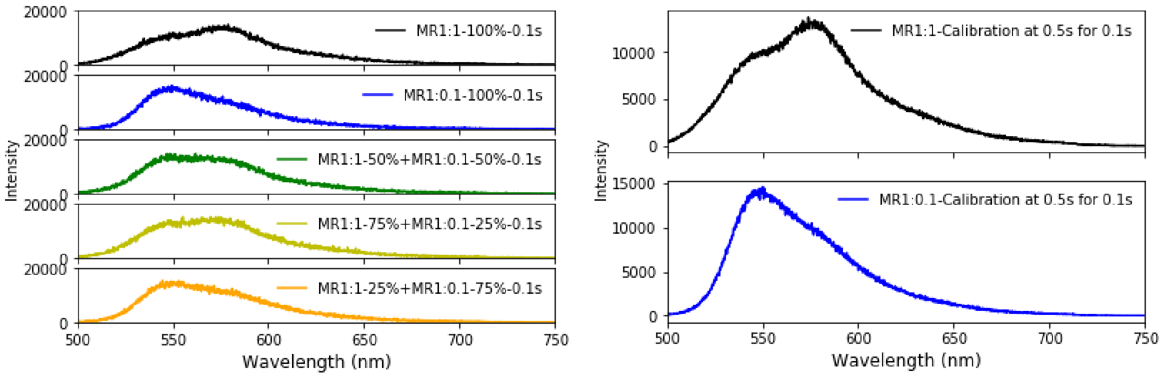

(**a**) (**b**)

Figure S3. Verification of multiplexing. (**a**) Fluorescence spectra collected with 100% of MR1:1 and MR1:0.1 and mix of 50+50, 75+25, 25+75% mix of MR1:1+MR1:0.1 particles respectively, excited at 488 nm. The time of collection of the fluorescence spectrum was 0.1 sec. (**b**) Normalized Spectra of pure MR1:1 and MR1:0.1 particles collected for 0.5 sec.

Table S4. Demultiplexing using different mixes of MR1:1 and MR1:0.1 particles excited at 488 nm and collected for 0.1sec.

Actual mix % MR1:1 : MR1:0.1 particles	Calculated mix % of MR1:1 : MR1:0.1	Error in demultiplexing MR1:1 / MR1:0.1	Average error in demultiplexing
100:0	97:7	3/7	5
25:75	27:82	2/7	4
50:50	48:61	2/11	6
75:25	76:38	1/13	7
0:100	-4:110	4/10	7

Table S5. Demultiplexing using different mixes of MR1:1 and MR1:0.1 particles excited at 488 nm and collected for 0.1sec with a weight function proportional to the fluorescent intensity.

Actual mix % MR1:1 : MR1:0.1 particles	Calculated mix % of MR1:1 : MR1:0.1	Error in demultiplexing MR1:1 / MR1:0.1	Average error in demultiplexing
100:0	100:4	0.4/4	2
25:75	28:81	3/6	5
50:50	52:59	2/9	5
75:25	79:36	4/11	7
0:100	-1:107	1/7	4

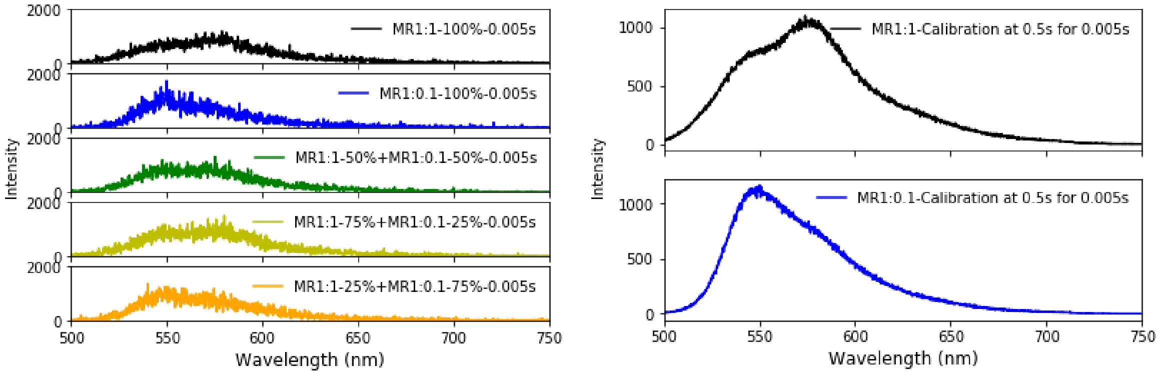

(**a**) (**b**)

Figure S4. Verification of multiplexing. (**a**) Fluorescence spectra collected with 100% of MR1:1 and MR1:0.1 and mix of 50+50, 75+25, 25+75% mix of MR1:1+MR1:0.1 particles respectively, excited at 488 nm. The time of collection of the fluorescence spectrum was 0.005 sec. (**b**) Normalized Spectra of pure MR1:1 and MR1:0.1 particles collected for 0.5 sec.

Table S6. Demultiplexing using different mixes of MR1:1 and MR1:0.1 particles excited at 488 nm and collected for 0.005sec.

Actual mix % MR1:1 : MR1:0.1 particles	Calculated mix % of MR1:1 : MR1:0.1	Error in demultiplexing MR1:1 / MR1:0.1	Average error in demultiplexing
100:0	72:7	28/7	18
25:75	9:74	16/0.7	9
50:50	30:50	20/0.3	10
75:25	57:33	18/8	13
0:100	-15:96	15/4	10

Table S7. Demultiplexing using different mixes of MR1:1 and MR1:0.1 particles excited at 488 nm and collected for 0.005sec with a weight function proportional to the fluorescent intensity.

Actual mix % MR1:1 : MR1:0.1 particles	Calculated mix % of MR1:1 : MR1:0.1	Error in demultiplexing MR1:1 / MR1:0.1	Average error in demultiplexing
100:0	81:2	19/2	10
25:75	17:71	8/4	6
50:50	41:44	9/6	8
75:25	68:27	7/2	4
0:100	-7:94	7/6	6

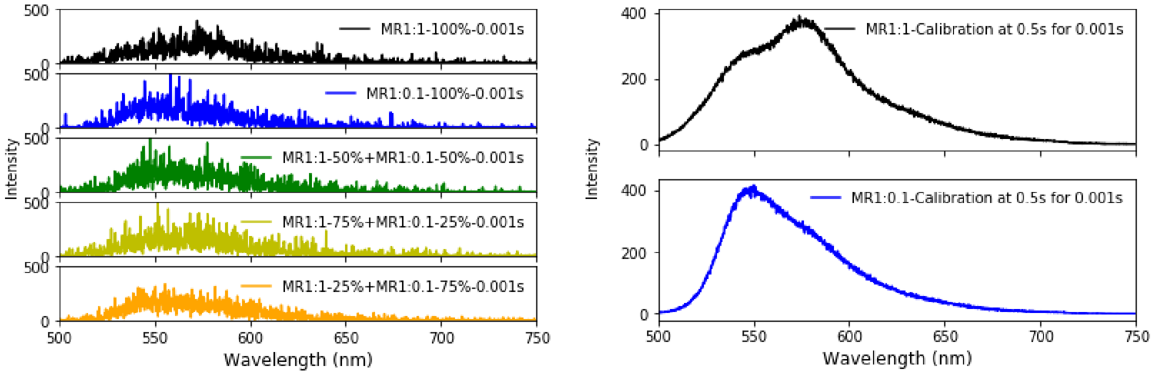

(**a**) (**b**)

Figure S5. Verification of multiplexing. (**a**) Fluorescence spectra collected with 100% of MR1:1 and MR1:0.1 and mix of 50+50, 75+25, 25+75% mix of MR1:1+MR1:0.1 particles respectively, excited at 488 nm. The time of collection of the fluorescence spectrum was 0.001 sec. (**b**) Normalized Spectra of pure MR1:1 and MR1:0.1 particles collected for 0.5 sec.

Table S8. Demultiplexing using different mixes of MR1:1 and MR1:0.1 particles excited at 488 nm and collected for 0.001sec.

Actual mix % MR1:1 : MR1:0.1 particles	Calculated mix % of MR1:1 : MR1:0.1	Error in demultiplexing MR1:1 / MR1:0.1	Average error in demultiplexing
100:0	43:2	58/2	30
25:75	7:39	18/36	27
50:50	10:37	40/13	26
75:25	31:17	45/8	26
0:100	-8:54	8/46	27

Table S9. Demultiplexing using different mixes of MR1:1 and MR1:0.1 particles excited at 488 nm and collected for 0.001sec with a weight function proportional to the fluorescent intensity.

Actual mix % MR1:1 : MR1:0.1 particles	Calculated mix % of MR1:1 : MR1:0.1	Error in demultiplexing MR1:1 / MR1:0.1	Average error in demultiplexing
100:0	57:-2	43/2	23
25:75	18:36	7/39	23
50:50	19:42	31/8	19
75:25	46:16	29/9	19
0:100	13:47	13/53	33

Table S10: Values of quantum yield of the dyes used in the paper:

Dye	Other name	QY
C504	C314	0.68[2]
R560	R110	0.91[3]
R6G		0.95[4]
RB		0.31[4]
R640	R101	0.913[5]
NB		0.27[6]

References

1. S. Palantavida, B. Peng and I. Sokolov, "Ultrabright fluorescent silica particles with a large number of complex spectra excited with a single wavelength for multiplex applications," *Nanoscale*, vol. 9, no. 15, pp. 4881-4890, 2017.
2. G. A. Reynolds and K. H. Drexhage, "New coumarin dyes with rigidized structure for flashlamp-pumped dye lasers," *Optics Communications*, vol. 13, no. 3, pp. 222-225, 1975.
3. S. P. Leytus, L. L. Melhado and W. F. Mangel, "Rhodamine-based compounds as fluorogenic substrates for serine proteinases," *The Biochemical journal*, vol. 209, no. 2, pp. 299-307, 1983.
4. D. Magde, G. E. Rojas and P. G. Seybold, "Solvent Dependence of the Fluorescence Lifetimes of Xanthene Dyes," *Photochemistry and Photobiology*, vol. 70, no. 5, pp. 737-744, 1999.
5. K. Rurack and M. Spieles, "Fluorescence Quantum Yields of a Series of Red and Near-Infrared Dyes Emitting at 600−1000 nm," *Analytical Chemistry*, vol. 83, no. 4, pp. 1232-1242, 2011.
6. R. Sens and K. H. Drexhage, "Fluorescence quantum yield of oxazine and carbazine laser dyes," *Journal of Luminescence*, vol. 24-25, pp. 709-712, 1981.
